# Comparing primary prevention with secondary prevention to explain decreasing Coronary Heart Disease death rates in Ireland, 1985–2000

**DOI:** 10.1186/1471-2458-7-117

**Published:** 2007-06-21

**Authors:** Zubair Kabir, Kathleen Bennett, Emer Shelley, Belgin Unal, Julia A Critchley, Simon Capewell

**Affiliations:** 1 Harvard School of Public Health, Division of Public Health Practice, Boston, USA; 2 Department of Pharmacology & Therapeutics, Trinity Centre for Health Sciences, St. James's Hospital, Dublin, Ireland; 3 Department of Health & Children, Hawkins House, Dublin, Ireland; 4 Department of Public Health, Dokuz Eylul University School of Medicine, Izmir, Turkey; 5 School of Population and Health Sciences, University of Newcastle upon Tyne, UK; 6 Department of Public Health, University of Liverpool, Liverpool, UK

## Abstract

**Background:**

To investigate whether primary prevention might be more favourable than secondary prevention (risk factor reduction in patients with coronary heart disease(CHD)).

**Methods:**

The cell-based IMPACT CHD mortality model was used to integrate data for Ireland describing CHD patient numbers, uptake of specific treatments, trends in major cardiovascular risk factors, and the mortality benefits of these specific risk factor changes in CHD patients and in healthy people without recognised CHD.

**Results:**

Between 1985 and 2000, approximately 2,530 fewer deaths were attributable to reductions in the three major risk factors in Ireland. Overall smoking prevalence declined by 14% between 1985 and 2000, resulting in about 685 fewer deaths *(minimum estimate 330, maximum estimate 1,285) *attributable to smoking cessation: about 275 in healthy people and 410 in known CHD patients. Population total cholesterol concentrations fell by 4.6%, resultingin approximately 1,300 (*minimum estimate 1,115, maximum estimate 1,660*) fewer deaths attributable to dietary changes(1,185 in healthy people and 115 in CHD patients) plus 305 fewer deaths attributable to statin treatment (45 in people without CHD and 260 in CHD patients). Mean population diastolic blood pressure fell by 7.2%, resulting in approximately 170 (*minimum estimate 105, maximum estimate 300*) fewer deaths attributable to secular falls in blood pressure (140 in healthy people and 30 in CHD patients), plus approximately 70 fewer deaths attributable to antihypertensive treatments in people without CHD.

Of all the deaths attributable to risk factor falls, some 1,715 (68%) occurred in people without recognized CHD and 815(32%) in CHD patients.

**Conclusion:**

Compared with secondary prevention, primary prevention achieved a two-fold larger reduction in CHD deaths. Future national CHD policies should therefore prioritize nationwide interventions to promote healthy diets and reduce smoking.

## Background

Coronary heart disease (CHD) remains the largest single cause of death in Ireland, as elsewhere in Europe, the USA and Australasia [1]. However, since the 1980s, CHD mortality rates have halved in Ireland, similar to many industrialised countries [1]. Studies in Europe, the USA and New Zealand consistently suggest that 50%–75% of the decrease in cardiac deaths can be attributed to population-wide improvements in the major risk factors, particularly smoking, total cholesterol and blood pressure [2-5]. The remaining 25%–50% of the decreased mortality fall [2-5] is generally explained by modern cardiology treatments for known CHD patients, such as thrombolysis, ACE inhibitors, statins, and coronary artery bypass surgery.

Consultants and department of health officials in particular prioritise risk factor reduction in CHD patients (secondary prevention), citing the low numbers needed to treat. However, epidemiological principles suggest that primary prevention (risk factor reduction in healthy subjects) may have a bigger potential than secondary prevention to reduce CHD deaths [6]. Although primary and secondary prevention interventions are probably both necessary to maximise population health, [4,6,7]quantifying their relative contributions is difficult using observational data [8]. Researchers have therefore used models to quantify the potential contribution of risk factor reductions before and after CHD manifests in an individual [2,8,9].

A better understanding of the relative contributions of primary prevention and secondary prevention to the recent decrease in CHD deaths is clearly very important. This would help to inform future CHD policy options in Ireland and elsewhere [10]. We have therefore used a validated and comprehensive CHD mortality model for Ireland to analyze the CHD mortality decrease between 1985 and 2000 [11]. We estimated the deaths avoided by changes in major cardiovascular risk factors in a) apparently healthy individuals ("primary prevention") and b) in patients with CHD ("secondary prevention") similar to a recent UK study [9].

## Methods

### The IMPACT CHD mortality model

The cell-based IMPACT CHD mortality model, previously validated in England and Wales [4], Scotland [3], New Zealand [5], Beijing [12], Finland [13], and now in Ireland [11], is described in detail on the IMPACT website [14]. In brief, the model was used to integrate data for the Irish population of 3.8 million, between 1985 and 2000. We attempted to include all individuals aged between 25 and 84 years, describing:

a) CHD patient numbers (ICD-9 code: 410–414), b) uptake and effectiveness of specific treatments, c) trends in major cardiovascular risk factors in apparently healthy subjects in populations and in specific patient groups, and d) the effectiveness (mortality benefits) of the reductions in specific risk factors in individuals with and without recognised CHD [11,14].

### Data sources

Information was obtained from routine health statistics, mainly the Public Health Information System (PHIS) [15], available from the Department of Health and Children, Ireland. Hospital Inpatient Enquiry (HIPE) data collected by the Economic and Social Research Institute were used though the system does not differentiate between first and recurrent events [16]. Prescription data were obtained from the General Medical Services (GMS) Payments Board and risk factor data mainly from two population studies, the Kilkenny Health Project and the Cork and Kerry Diabetes and Heart Disease Study [17,18], as well as from the Central Statistics Office [19]. Data on the effectiveness of therapeutic interventions and the mortality reduction from specific population cardiovascular risk factor changes came from recent published meta-analyses randomised controlled trials, and cohort studies [20-22]. Further details on the Irish data sources are in appendices 1–3 [see Additional file 1].

### Primary prevention estimates in people without recognized CHD

The Irish IMPACT CHD Mortality model calculated the numbers of CHD deaths prevented/postponed using a similar methodology recently undertaken in England and Wales [9]. For risk factor changes, the model uses regression (β) coefficients obtained from large meta-analyses and cohort studies [13,22,23]. The details are shown in Appendix 4 [see Additional file 1]. Given the absence of suitable β coefficients for obesity, diabetes, and physical activity, relative risk estimates were used. However, for the present study only the three main cardiovascular risk factors (cholesterol, smoking and blood pressure) were considered, along with hypertension and statin treatments. All the regression coefficients and relative risk values were obtained from multivariate logistic regression analyses and were therefore assumed to be independent, having adjusted for potential confounding from other major risk factors [13,22,23].

Each β coefficient quantifies the independent relation between population change in a specific CHD risk factor (such as smoking, cholesterol, or blood pressure)and the consequent percentage change in population mortality from CHD. We then estimated the subsequent reduction in the number of deaths produced by the decrease in each major risk factor as the product of three variables: the number of CHD deaths observed in the base year (1985), the relative reduction in that risk factor, and the β coefficient, each stratified by age and sex [11,14].

To estimate the impact of the population-wide reduction in cholesterol due to dietary change, we subtracted the estimated effect of statins for primary prevention from the overall number of deaths prevented or postponed in the population due to change in mean cholesterol concentration. We explicitly considered demographic change by using age and sex specific population CHD mortality and CHD patient numbers for 1985 and for 2000 [11].

### Secondary prevention estimates in CHD patients

The mortality benefit attributable to reductions in each major risk factor (smoking, total cholesterol, and blood pressure) in each group of CHD patients were estimated as the number of deaths prevented or postponed similar to the calculation for effects on primary prevention. However, for secondary prevention in CHD patients, we used relative risks for the three main risk factors studied which were different from the beta coefficients employed for primary prevention estimates. The estimates used for secondary prevention are relatively low than those given in Appendix 4 [see Additional file 1] that were used for primary prevention estimates. We estimated the number of deaths prevented or postponed for specific age and sex groups. We used survival benefit over a one year time interval throughout.

We categorised CHD patients according to disease groups: acute myocardial infarction, survivors of myocardial infarction, revascularisation patients, and patients with unstable angina, chronic angina, and chronic heart failure. We did not consider the impact of changes in risk factors in patients with an initial acute myocardial infarction or unstable angina, because both are transient states. To avoid double counting, we firstly made adjustments for overlaps between different treatment groups by subtracting the overlapping subgroup from the main group [11,14].

We based age and sex specific smoking cessation rates on local surveys and audits. We initially assumed that age and sex specific changes in cholesterol attributable to diet and changes in blood pressure attributable to population secular trends would mirror the changes seen in the general population. We then used rigorous sensitivity analyses to test the effect of much smaller (-50%) and much larger (+50%) changes.

### Statins and other treatments

The model aimed to include all medical and surgical treatments provided in 2000. This included statins as primary prevention (in people without recognised CHD) and as secondary prevention(in CHD patients). We calculated the absolute reduction in mortality by using the relative reduction in mortality reported in the most recent meta-analysis [20] applied to the age specific case fatality rate observed in unselected patient cohorts. The effect of all other "secondary prevention" drugs (aspirin, β blockers, and angiotensin converting enzyme inhibitors) has been previously reported and was explicitly excluded from this analysis [11]. We did not consider over the counter statins, as these only became available in 2003.

### Sensitivity analysis

Because of the uncertainties surrounding some values, we did a multiway sensitivity analysis using the analysis of extremes method [24].

### Apportioning deaths prevented or postponed between primary and secondary prevention

We then estimated the deaths prevented or postponed in apparently healthy people as the deaths prevented or postponed in the entire population minus the deaths prevented or postponed in each CHD patient group.

## Results

### CHD mortality declines between 1985 and 2000 due to risk factor reductions

Between 1985 and 2000, about 2,530 fewer deaths were attributable to reductions in the three major cardiovascular risk factors in Ireland. Approximately 1,715 deaths *(minimum estimate 1,195, maximum estimate 2,370) *were prevented or postponed due to decreases in the three major risk factors: smoking, cholesterol and blood pressure among apparently 'healthy individuals' (primary prevention); that includes both statin (45 fewer deaths) and hypertension therapy (70 fewer deaths) (Table 1). A further 815 deaths (*minimum estimate 500, maximum estimate 1,635) *were attributed to changes in these three risk factors in recognized CHD patients (secondary prevention), including 260 fewer deaths due to statin therapy.

**Table 1:** The CHD mortality fall in Ireland attributable to risk factor changes in individuals with and without recognised coronary heart disease, including sensitivity analyses (minimum and maximum estimates)

	**Relative change in population risk factor level (%)**	Deaths prevented or postponed * (minimum and maximum estimates)		
		
		**In healthy subjects (Primary prevention)**	**In CHD patients (Secondary Prevention)**	**TOTALS**
**SMOKING Total Change**	**-14.2%**	**275 **(20–485)	**410 **(310–800)	**685 **(330 – 1,285)
**CHOLESTEROL Total change**	**-4.6%**	**1,230 **(1060–1,520)	**375 **(170–770)	**1,605 **(1,230 – 2,290)
***Fall Attributable to diet***		***1,185 ****(1050–1,445)*	***115 ***(65–215)	***1,300 ****(1,115–1,660)*
***Fall Attributable to statins***		***45 ***(10–75)	***260 ****(105–555)*	***305 ****(115–630)*
**BLOOD PRESSURE Total Change**	**-7.2%**	**210 **(115–365)	**30 **(20–65)	**240 **(135–430)
***Secular trends***		***140 ***(85–235)	***30 ***(20–65)	***170 ****(105–300)*
***Hypertension therapy***		***70 ***(30–130)	**	***70 ***(30–130)

**All 3 major risk factors**		**1,715(68%) **(1,195–2,370)	**815(32%) **(500–1,635)	**2,530 **(1,695–4,005)

*All numbers were rounded to the nearest 5;

**Hypertension therapy in CHD patients already quantified within the secondary prevention medication component of the IMPACT Model.

Overall smoking prevalencedeclined by 14% between 1985 and 2000, resulting in about 685 fewer deaths *(minimum estimate 330, maximum estimate 1,285) *attributable to smoking cessation: about 275 in healthy people and 410 in known CHD patients. Population total cholesterol concentrations fell by 4.6%, resulting in approximately 1,300 (*minimum estimate 1,115, maximum estimate 1,660*) fewer deaths attributable to dietary changes (1,185 in healthy people and 115 in CHD patients) plus 305 fewer deaths attributable to statin treatment (45 in people without CHD and 260 in CHD patients). Mean population diastolic blood pressure fell by 7.2%, resulting in approximately 170 (*minimum estimate 105, maximum estimate 300*) fewer deaths attributable to secular falls in blood pressure (140 in healthy people and 30 in CHD patients), plus approximately 70 fewer deaths attributable to antihypertensive treatments in people without CHD.

Of all the deaths attributable to risk factor falls, some 1,715 (68%) occurred in people without recognized CHD and 815 (32%) in CHD patients.

The relative contribution to the overall decline in CHD deaths from primary and secondary prevention for each risk factor was little changed whether considering best, minimum, or maximum estimates (sensitivity analyses) (Table 1). A similar pattern was also observed in both men and women across all age-groups (Table 2). However, the overall contribution from primary prevention was consistently higher in all age groups for both sexes (Table 2).

**Table 2:** Numbers of deaths prevented or postponed by risk factor reductions:Relative contributions from primary prevention and secondary prevention, by age and sex

	*AGE*	*GROUPS*	*TOTAL*
MEN	45–64 years	65–84 years	

Risk Factor TOTAL	585	1,030	1,615**
Secondary Prevention	190	325	515
Primary Prevention	395	705	1,100
			
Secondary prevention (%)	32%	32%	32%
Primary prevention (%)	68%	68%	68%

			

	AGE	GROUPS	TOTAL

WOMEN	45–64 years	65–84 years	

Risk Factor TOTAL	145	725	870
Secondary Prevention	55	245	300
Primary Prevention	90	480	570
			
Secondary prevention (%)	38%	34%	34%
Primary prevention (%)	62%	66%	66%

*All numbers rounded to the nearest 5

**In 25–44 year-olds only 45 fewer CHD deaths occurred, and therefore not shown in this table.

## Discussion

More than two-thirds of the large reduction in CHD deaths in Ireland between 1985 and 2000 may be attributed to primary prevention, defined here as reductions in the three major risk factors (smoking, cholesterol and blood pressure) in individuals without recognised CHD. Furthermore, primary prevention had more than two-fold greater impact than secondary prevention (defined here as risk factor reductions in recognised CHD patients). This is much as predicted by Rose [6], and others [8]. A recent study in Ireland also showed that twice as many life-years were generated by relatively modest reductions in the three major cardiovascular risk factors when compared with the use of modern cardiology treatments [25].

Reductions in the three major cardiovascular risk factors (cholesterol, smoking and blood pressure) between 1985 and 2000 accounted for about 2,530 fewer deaths in Ireland in 2000. The two biggest contributions came from large decreases in smoking prevalence and cholesterol levels. This resulted in about 2,290 fewer deaths, of which about 1,505 were in "healthy subjects", while approximately 410 and 115 fewer deaths resulted from smoking cessation and from diet-based cholesterol reductions in CHD patients, respectively and the remainder from blood pressure reductions. Abramson and Wright, in a meta-analysis of randomised trials in a primary prevention population, found that statin therapy did not reduce total CHD events in women or in all those over 69 years of age [26]. Instead, modification of lifestyles to reduce cholesterol was considered more acceptable, as shown in our study, by the greater benefits of diet change in the primary prevention population in reducing cholesterol. In contrast, there were greater benefits observed in reducing cholesterol, by prescribing statins in the secondary prevention population. This contrasting pattern is likely to reflect the differences in cardiovascular risk and potential benefits of treatment versus lifestyle changes in those with and without CHD.

Opportunities for secondary prevention are frustratingly limited, because some 50% of myocardial infarctions are rapidly fatal [27]. This emphasises the importance of primary prevention. It makes sense to energetically target smokers *before *they develop clinical disease. In Ireland, the National Workplace Smoking Ban introduced in March 2004 has already achieved significant improvements in smoking prevalence and in air quality [28,29].

Irish population levels of both cholesterol and blood pressure declined between 1985 and 2000 [11]. The factors underlying these declines are clearly complex. Having explicitly considered all the cardiology therapeutic interventions in our main IMPACT CHD mortality model [9,11], the residual decline in total cholesterol levels might reasonably be attributed to dietary changes [30]. Reduced intake of saturated fat and salt will also have benefited population blood pressure [31]. Recent improvements in physical activity levels were, however, minor [32]. Randomised trials demonstrate that it is hard to achieve substantial and sustained changes in dietary habits in individuals [33,34]. Substantial falls in average total cholesterol have been achieved in entire populations including Finland, Mauritius and Poland [30,35,36]. These falls almost certainly reflect a combination of factors: national policy, economics, health promotion and multisectoral collaboration, as well as advice to individuals [30,35,36]. International evidence is thus increasingly powerful.

The most effective interventions to reduce major risk factors have come from comprehensive cardiovascular strategies underpinned by robust national policies [35,36]. The 1999 Irish Cardiovascular Health Strategy is comprehensive, outlining a wide range of recommendations, including a secondary prevention programme [10]. Such programmes appear cost-effective [8,34].

Our study findings also show that preventative approaches do not have a sex difference in preventing or postponing CHD deaths in Ireland (Table 2). The majority of such preventable deaths occurred among the oldest age-groups (65–84 years). However, further reductions in preventable CHD deaths could be achieved in Ireland and elsewhere if potentially younger individuals (both apparently healthy and at risk) are also targeted. The relatively low contribution to fewer CHD deaths through secondary prevention in this study also highlights the importance of strengthening secondary prevention programmes in Ireland.

The study findings strongly support the population prevention approach [6,8]. Over 20 years ago, Rose clearly demonstrated that apparently small changes across the entire population would achieve far larger overall mortality gains than much greater changes in a relatively small number of high risk individuals [6]. The evidence base underlying the IMPACT CHD mortality model is now strengthened by several recent studies [10,20,23].

### Limitations of the study

All modelling analyses should be interpreted with appropriate caution. All require numerous data inputs, each with recognized limitations. We therefore sometimes had to use data from studies possibly constrained by geographic or selection bias, or extrapolate to older age groups, or make explicit assumptions. However, our rigorous analysis of extremes approach suggested that the proportional contributions to the overall reductions in deaths from primary versus secondary prevention remained reasonably consistent, irrespective of whether best, minimum, or maximum estimates were considered. This was reassuring, as was the general consistency with studies performed elsewhere [3-5,13]. Furthermore, we only considered the three main risk factors in this study although the IMPACT CHD mortality model does account for the remaining risk factors (obesity, diabetes and physical inactivity levels) and also for all the cardiology treatments [9,11,25]. Specific parameters such as LDL/HDL or TC/HDL ratios were not considered. This merits further work.

## Conclusion

In conclusion, more than two-thirds of the recent large falls in CHD deaths in Ireland can be attributed to improvements in the levels of the three main cardiovascular risk factors. Much as predicted, primary prevention had a two-fold bigger mortality impact than risk factor reduction in CHD patients. Comprehensive CHD strategies should emphasize that primary prevention is also cost-effective. Tobacco control, healthier diets and physical activity should therefore be prioritised in future national CHD strategies, especially targeting relatively young populations. Moreover, further reductions in CHD deaths could be achieved if secondary prevention programmes were also strengthened.

## Competing interests

The author(s) declare that they have no competing interests.

## Authors' contributions

ZB and KB developed the Irish epidemiology model and drafted the manuscript. ES conceived original idea and helped draft the manuscript. BU, JC and SC were involved in the development of the original model and its application to Irish data. All authors read and approved the final manuscript.

## Pre-publication history

The pre-publication history for this paper can be accessed here:



## Supplementary Material

Additional File 1 Appendix 1 – Populations statistics and patient eligibility data sources for Ireland: 1985–2000. Click here for file
