# Supplementary material for: Comparing primary prevention with secondary prevention to explain decreasing Coronary Heart Disease death rates in Ireland, 1985–2000
Source: BMC Public Health. 2007 Jun 21;7:117. doi: 10.1186/1471-2458-7-117 (PMC1914046; doi:10.1186/1471-2458-7-117)
Supplement: Additional File 1 — Appendix 1 – Populations statistics and patient eligibility data sources for Ireland: 1985–2000. [file 1471-2458-7-117-S1.doc]

**Appendix 1: Population statistics and patient eligibility data sources for Ireland: 1985-2000**

Information Source

Population Statistics Central Statistics Office ([www.cso.ie](http://www.cso.ie/)); Public Health Information System (PHIS)^1^

Deaths by age and sex Central Statistics Office ([www.cso.ie](http://www.cso.ie/)); PHIS^1^

CHD Mortality (Rates) PHIS^1^

Hospital Myocardial Infarction Patients PHIS^1^; Hospital In-Patient Enquiry (HIPE) ^2^

Hospital Angina and Heart Failure Patients HIPE^1^

Hospital CABG Surgery Patients Irish Cardiac Surgery Register^3^; PHIS^1^

Hospital Angioplasty Patients Irish Cardiac Surgery Register^3^; PHIS^1^

CPR Patients Irish Cardiac Surgery Register^3^; Coronary Care Census (2003)^4^

Secondary Prevention Therapies PHIS^3^; Coronary Care Census (2003) ^4^

Angina Patients in Community General Medical Services (GMS) Payments Board ([www.gmspb.ie](http://www.gmspb.ie/))

Hypertension Patients in Community Kilkenny Health Project^5^; Cork and Kerry Diabetes and Heart Disease Study^6^

Community Heart Failure Patients General Medical Services (GMS) Payments Board ([www.gmspb.ie](http://www.gmspb.ie/))

^1^ Information management unit Department of Health and Children. Public Health Information System (PHIS). [6], Ireland. 2001.

^2^  HIPE and NPRS Unit. *Activity in Acute Public Hospitals in Ireland 1990-1999*. Dublin: The Economic and Social Research Institute, 2002.

^3^ Lonergan M on behalf of the Irish Cardiac Surgery Group. *Irish Cardiac Surgery Register Report 1983-200.* Dublin*:* Department of Health and

Children, 2002.

^4^  Doyle F, De La Harpe D, McGee H, Shelley E, Walsh M, Daly K. Nine-year comparison of presentation and management of acute coronary

syndromes in Ireland: a national cross-sectional survey. *BMC Cardiovasc Disord* 2005; 5: 5.

^5^ Shelley E and working group. The Kilkenny Health Project: A community Research and Demonstration Programme for Cardiovascular disease

prevention. *Ir J Med Sci* 1991; 160 (Supplement 9): 1-54.

^6^ Creagh D, Neilson S, Collins A, Colwell N, Hinchion R, Drew C et al. Established cardiovascular disease and CVD risk factors in a primary

care population of middle-aged Irish men and women. *Ir Med J* 2002; 95(10):298-301.

**Appendix 2: Data sources on cardiovascular risk factors in Ireland, 1985-2000.**

**Cardiovascular Risk Factors Source: Initial year (1985) Source: Most recent year (2000)**

Population Blood Pressure Belfast MONICA mid-data ^1^ Cork and Kerry Diabetes and Heart Disease Study^2^

Smoking Prevalence Central Statistics Office Survey of Lifestyle, Attitudes & Nutrition (SLAN) ^3^

([www.cso.ie](http://www.cso.ie/))

Cholesterol Belfast MONICA mid-data ^1^ Cork and Kerry Diabetes and Heart Disease Study^2^

(for 25-34 year-olds)

Kilkenny Health Project^4^

(for 35 year-olds and above)

Diabetes Kilkenny Health Project^4^ General Medical Services (GMS) Payments Board

([www.gmspb.ie](http://www.gmspb.ie/));

Cork and Kerry Diabetes and Heart Disease Study^2^

Obesity Kilkenny Health Project^4^ SLAN ^3^; Irish Universities Nutrition Alliance ^5^;

Cork and Kerry Diabetes and Heart Disease Study^2^

Physical Activity No Irish data SLAN ^b^; Irish Universities Nutrition Alliance ^5^

^1^ Evans AE *et al*. Coronary risk factor prevalence in a high incidence area: results from the Belfast MONICA Project. *Ulster Med J* 1989; 58: 60-8.

^2^ Creagh D, Neilson S, Collins A, Colwell N, Hinchion R, Drew C et al. Established cardiovascular disease and CVD risk factors in a primary

care population of middle-aged Irish men and women. *Ir Med J* 2002; 95(10):298-301.

^3^ Department of Health & Children. *The National Health & Lifestyle Surveys*. Dublin: Centre for Health Promotion Studies, April 2003.

^4^ Shelley E and working group. The Kilkenny Health Project: A community Research and Demonstration Programme for Cardiovascular disease

prevention. *Ir J Med Sci* 1991; 160 (Supplement 9): 1-54.

^5^ Irish Universities Nutrition Alliance. *North/South Ireland food consumption survey: food and nutrient intakes, anthropometry, attitudinal data and physical*

*activity patterns.* Dublin: Food Safety Promotion Board, 2001.

**Appendix 3. Irish data sources for treatment uptake levels in 2000**

Treatments Treatment uptake^1^ Source

**Acute MI**

Thrombolysis 44% (age & gender gradient) Coronary Care Census (2003)^2^

Aspirin 56% (age & gender gradient) EUROASPIRE II ^3^

Primary angioplasty 4% Coronary Care Census (2003)^2^

Beta-blockers 33% EUROASPIRE II ^3^

ACE inhibitors 20% (age & gender gradient) EUROASPIRE II ^3^

**Secondary Prevention**

Aspirin 85% (age &gender gradient) Coronary Care Census (2003)^2^

Beta-blockers 47% (age &gender gradient) EUROASPIRE II ^3^

ACE inhibitors 27% (age &gender gradient) EUROASPIRE II ^3^

Statins 62% (age & gender gradient) EUROASPIRE II ^3^

Warfarin 4% EUROASPIRE II ^3^

Rehabilitation 54% EUROASPIRE II ^3^

**Chronic Angina**

Aspirin (Community) 75% (age & gender gradient) GMS ([www.gmspb.ie](http://www.gmspb.ie/))

Statins (Community) 75% (age & gender gradient) GMS ([www.gmspb.ie](http://www.gmspb.ie/))

**Heart Failure**

**(Community)**

ACE inhibitors No Irish data

Beta blockers 22% (age & gender gradient) GMS ([www.gmspb.ie](http://www.gmspb.ie/))

Spironolactone 7% (age & gender gradient) GMS ([www.gmspb.ie](http://www.gmspb.ie/))

Aspirin 56% (age & gender gradient) GMS ([www.gmspb.ie](http://www.gmspb.ie/))

Statins 7% (age & gender gradient) GMS ([www.gmspb.ie](http://www.gmspb.ie/))

**Hypertension** 38% (age & gender gradient) Cork and Kerry Diabetes and Heart

Disease Study^4^

**Statins for Primary** 6% (age & gender gradient) GMS ([www.gmspb.ie](http://www.gmspb.ie/))

**Prevention**

^1^ The % values are the maximum observed uptakes based on the sources alongside, but

they are weighted to age and gender groups in model estimates.

^2^  Doyle F, De La Harpe D, McGee H, Shelley E, Walsh M, Daly K. Nine-year comparison of

presentation and management of acute coronary syndromes in Ireland: a national cross-

sectional survey. *BMC Cardiovasc Disord* 2005; 5: 5.

^3^ EUROASPIRE II Study Group. *Eur Heart J* 2001; 22: 554-72.

^4^ Creagh D, Neilson S, Collins A, Colwell N, Hinchion R, Drew C *et al*. Established

cardiovascular disease and CVD risk factors in a primary care population of middle-aged men and women. *Ir Med J* 2002; 95(10):298-301.

**Appendix 4: Sources of beta coefficients and relative risk estimates for risk factors**

**Risk factors Beta coefficients (absolute)**

**Men Women**

Smoking^1^ 0.81 1.24

Blood Pressure^1^ 0.02 0.03

Cholesterol^2^ 0.63 0.52

**Relative Risks (95% confidence interval)**

**Men Women**

Obesity^3^ 2.24 (2.03 – 2.47) 2.26 (1.90 – 2.68)

Diabetes^3^ 2.67 (2.36 – 3.02) 4.26 (3.51 – 5.18)

Physical activity^3^ 0.77 (0.69 – 0.85) 0.48 (0.39 – 0.59)

^1^ Laatikainen T, Critchley J, Vartiainen E, et al. Explaining the decline in coronary heart

disease mortality in Finland between 1982 and 1997. *Am J Epidemiol* 2005;162: 764-

773.

^2^ Law MR, Wald NJ, Thompson SG. By how much and how quickly does reduction in serum cholesterol concentration lower risk of ischaemic heart disease? *BMJ* 1994; 308:367-72.

^3^ [Yusuf S, Hawken S, Ounpuu S, Dans T, Avezum A, Lanas F, McQueen M, Budaj A, Pais P, Varigos J, Lisheng L; INTERHEART Study Investigators.](http://www.ncbi.nlm.nih.gov.ezp1.harvard.edu/entrez/query.fcgi?holding=hulib&db=pubmed&cmd=Retrieve&dopt=AbstractPlus&list_uids=15364185&query_hl=8&itool=pubmed_docsum) Effect of potentially modifiable risk factors associated with myocardial infarction in 52 countries (the INTERHEART study): case-control study. *Lancet* 2004; 364:937-52
